# A Long-Term Monitoring Method of Corrosion Damage of Prestressed Anchor Cable

**DOI:** 10.3390/mi14040799

**Published:** 2023-03-31

**Authors:** Jianzhi Li, Chen Wang, Yiyao Zhao

**Affiliations:** 1 Structural Health Monitoring and Control Institute, Shijiazhuang Tiedao University, Shijiazhuang 050043, China; 2 School of Mechanical Engineering, Shijiazhuang Tiedao University, Shijiazhuang 050043, China; 3 School of Materials Science and Engineering, Shijiazhuang Tiedao University, Shijiazhuang 050043, China

**Keywords:** prestressed anchor cable, axial-distributed optical fiber, corrosion monitoring

## Abstract

Based on high-stress characteristics of prestressed anchor cables, this paper develops an axial-distributed testing method to test corrosion damage of prestressed anchor cables. The positioning accuracy and corrosion range of an axial-distributed optical fiber sensor is studied, and its mathematical model between corrosion mass loss and axial fiber strain is established. The experimental results show that the fiber strain from an axial-distributed sensor enables one to reflect the corrosion rate along a prestressed anchor. Moreover, it has a greater sensitivity when an anchored cable has a higher stress. The mathematical model between corrosion mass loss and axial fiber strain is determined to be ε=4723.64ρ+2592.95. The corrosion location along the anchor cable is characterized by axial fiber strain. Therefore, this work provides an insight for cable corrosion.

## 1. Introduction

Prestressed anchorage technology has been widely used in engineering. However, corrosion can randomly occur along a prestressed cable, and in turn lead to its failure. Therefore, corrosion monitoring of prestressed anchor cables is of great significance to structural safety. In recent years, optical fiber sensing technology has attracted much attention in the field of reinforcement corrosion monitoring due to its incomparable advantages such as small size, soft winding, ease of compositing with structure, corrosion resistance, and distributed measurement.

The measurement methods of environmental parameters such as step-type corrosion fiber sensor [1,2,3], long-period fiber grating (LPFG) refractive index sensitive sensor [4], and fiber Bragg grating (FBG) refractive index sensitive sensor [5,6] are commonly used in this field. However, it enables one to indirectly reflect the corrosion of reinforcement. For example, Gan Wei Zhong from Zhejiang University [1,2,3] designed a step-type corrosion fiber sensor to measure the position change of a concrete blunt front surface because concrete is gradually blunted before steel reinforcement corrosion and, moreover, its blunt front surface moves towards steel reinforcement with corrosion time. Liu Hong Yue et al. [4] designed a LPFG steel corrosion sensor based on the refractive index magnitude of the ambient concrete around the reinforcement. Muhammad [5] and Tan [6] used HF acid to corrode FBG cladding and acquire the response relationship between FBG wavelength and its external refractive index. The trapezoidal sensor reflects steel corrosion by testing the blunt front surface of concrete. Although both LPFG and FBG refractive index sensors can measure the corrosion damage of steel bars, the complex packaging process seriously affects its long-term reliability and is susceptible to environmental interference.

On the other hand, the corrosion measurement of reinforcement also depends on the expansion of reinforcement corrosion products using fiber grating sensors and fully distributed optical fiber sensors. For example, some scholars have proposed a Fe-C film FBG sensor [7,8,9], circumferential layout FBG sensor [10], double reinforcement corrosion FBG sensor [11], FBG strain sensor [12], LPFG bending sensor [13], and other methods, and have measured the uniform corrosion of reinforcement based on the above-mentioned principle. However, these methods are difficult to apply in practical engineering. In addition, due to the poor affinity between Fe-C film and quartz glass, its service lifetime is seriously shorted, and its performance is deteriorated.

Simultaneously, the fully distributed optical fiber sensing system uses an optical fiber as both the sensing element and signal transmission medium to detect the changes of temperature and strain along different positions of optical fiber in order to realize a truly distributed measurement. Zhao Xuefeng designed a white light interference corrosion sensor [14], a Brillouin distributed optical fiber optic sensor [15], and a low coherence fiber strain sensor [16], and predicted the corrosion by testing light intensity and frequency shift. Among them, the white light interference corrosion sensor and Brillouin distributed optical fiber optic sensor has a prominent feature of being tightly wound on the steel bar or mortar cover to form fiber coil. Hence, a local extrusion from the increase of rust expansion strain often occurs and causes a deteriorated signal-to-noise ratio of Brillouin signal with the increasing corrosion time, which enable one to attain an early-stage measurement of steel bar corrosion instead of a later measurement. Furthermore, a low-coherence fiber-optic strain sensor based on the Michelson interference principle has been proposed to measure the later corrosion when the structural deformation exceeds 1000 uɛ or more. At the same time, Mao Jianghong also proposed a ring sensor based on the principle of rust expansion [17,18,19]. However, due to the limitation of spatial resolution and the sensor package, these above-mentioned sensors are discrete devices and fail to attain fully distributed measurements. Additionally, they fail to locate corrosion damage. Furthermore, a spiral wound distributed sensor [20,21] has been proposed to achieve a fully distributed measurement. However, this method causes light loss due to its spirally wound structure and in turn causes a deteriorated signal-to-noise ratio.

Therefore, to decrease light loss and improve the signal-to-noise ratio, this paper proposes an axial-distributed measurement method. The mathematical model of corrosion mass loss rate and axial-distributed optical fiber strain is ultimately established, as well as a long-term monitoring method of corrosion damage of prestressed anchor cable, which enable one to solve the "bottleneck" of corrosion monitoring methods.

## 2. Theoretical Analysis

Prestressed anchor cables are characterized by a high-tension stress. The corrosion of prestressed anchor cable eventually leads to a reduction in cross-sectional area. Correspondingly, the strain along anchor cables with a high prestress increases at the corrosion site. Equation (1) shows the strain is dependent on its cross-sectional area.

(1) $$\varepsilon =\frac{F}{ES}$$

Therefore, the distributed strain along the anchor cable indicates its corrosion. Furthermore, the strain difference caused by cable corrosion under high-stress state is deduced:

(2) $$\Delta \varepsilon =\frac{F}{E}\left(\frac{1}{S}-\frac{1}{S_{0}}\right)$$

Corrosion of prestressed anchor cable causes the section loss of strand steel, and the section loss rate is equal to its theoretical mass loss rate ρ. The mathematical relation of theoretical corrosion mass loss rate is expressed as follows:

(3) $$\rho =\eta =\frac{m_{0}-m}{m_{0}}=\frac{\rho LS_{0}-\rho LS}{\rho LS_{0}}=\frac{S_{0}-S}{S_{0}}$$

Then, Equation (3) is derived:

(4) $$\rho =\frac{S_{0}-S}{S_{0}}=\frac{\frac{F}{E*\varepsilon_{0}}-\frac{F}{E*\varepsilon }}{\frac{F}{E*\varepsilon_{0}}}=\frac{\frac{1}{\varepsilon_{0}}-\frac{1}{\varepsilon }}{\frac{1}{\varepsilon_{0}}}=1-\frac{\varepsilon_{0}}{\varepsilon }$$

Subsequently, the cable strain is expressed as

(5) $$\varepsilon =\frac{F}{ES_{0}\left(1-\rho \right)}$$

where ρ is the cross-section loss rate, which is equal to the mass loss rate; F is the initial prestress; E is its elastic modulus; $\varepsilon_{0}$ is its strain without corrosion;ε is its strain after corrosion; S is its cross-sectional area; $S_{0}$ is its initial cross-sectional area without corrosion; and Δε is the corrosion-induced strain difference compared to the initial strain without corrosion.

In the practical engineering, the tensile force of a monofilament strand with a 5 mm diameter is approximately 24 kN, its elastic modulus approximately 200 GPa, and its initial cross-sectional area is 19.625 mm^2^. Substituting the above data into Equation (5), the relation between cable strain and corrosion loss rate is obtained. Cable strain is attained by an axial-distributed sensor. Hence, it measures the corrosion mass loss rate using the distributed strain along the anchor cable.

**Figure 1:**
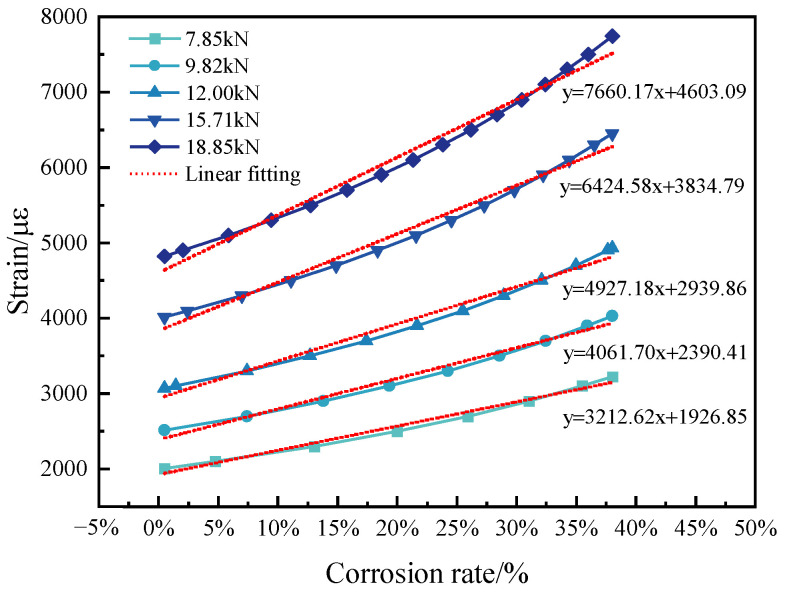
The relation between strain and corrosion mass loss rate.

Figure 1 shows the strain response to the theoretically calculated corrosion rate. From this figure, we see that the strain basically increases linearly with corrosion rate. When the corrosion rate ranges from 0.48% to 38%, the slope of linear fitting depends on the initial tension stress. When the initial stress of the anchored cable is 400 MPa, the slope of the curve is 3212.62, while the slope of a 960 MPa initial strain is 7660.17. These findings indicate that the axial sensor has a greater sensitivity when an anchored cable has a higher stress.

## 3. Materials and Methods

**Figure 2:**
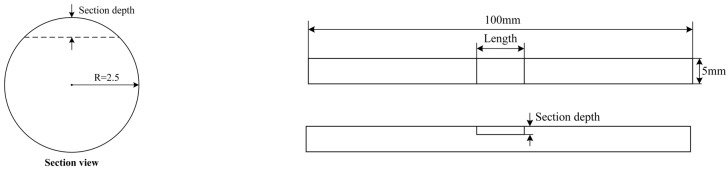
Specimen diagram.

**Figure 3:**
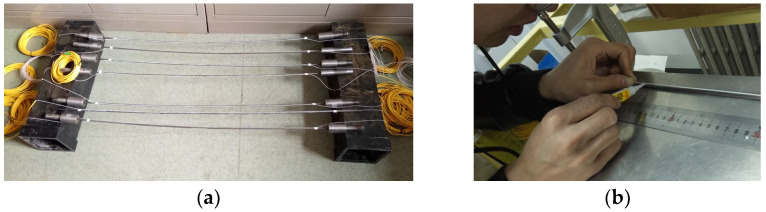
The photograph of the specimens used: (**a**) the manufactured specimen; (**b**) glued optical sensing fiber.

To establish a mathematical model of the axial strain and the corrosion mass loss rate of prestressed anchor cable, a tensile test was conducted for different specimens with a variety of sectional losses. The specimens with different sectional losses correspond to different corrosion mass losses. The strand dimensions used were 5 mm in diameter and 1 m in length. The tensile strength was 1860 MPa and the elastic modulus was approximately 200 GPa. The graded loading method was used in this experiment. There were 1#, 2#, 3#, 4#, 5#, 6#, 7# specimens with a reduced sectional area fabricated to simulate different corrosion loss rates and corrosion lengths (Figure 2). The section depth and length are shown in Table 1. The details of these specimens are given in Figure 3. The MTS hydraulic servo material testing machine was used, and an increasing tension was applied step by step. The maximum stress applied in the experiments was 1228 MPa. Specimens 1#, 2#, 3#, 4#, 5#, 6#, and 7# were stretched with a graded load. The graded load was 2 kN and the maximum load was 22 kN, 22 kN, 22 kN, 20 kN, 18 kN, 16 kN, and 14 kN, respectively.

In order to ensure full contact between the optical fiber and the strand, and to avoid errors caused by strain transfer, the strand was first polished with sandpaper to make its surface smooth. In order to prevent the impact of grinding scraps, stains, etc., alcohol was used clean the impurities. A 0.9 mm tight optical sensing fiber was glued with AB epoxy glue along the steel strand afterwards. These optical fiber sensors have a higher strain transfer. The specimens are shown in Figure 3.

**Figure 4:**
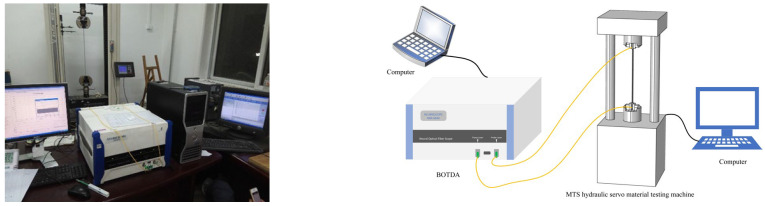
Schematic diagram of experiment.

An axial-distributed optical fiber sensor was used to simultaneously monitor the stress and strain of the prestressed steel strand specimens. The PPP-BOTDA measures spatially distributed strains along the optical fiber through measuring the time of flight of light from Brillouin backscattering [22,23,24]. The operating principle of PPP-BOTDA is similar to that of BOTDA [15]. The Brillouin frequency shift ($\Delta v_{B}$) is related to the temperature change (ΔT) and strain change (Δε) [25]:

(6) $$\Delta v_{B}=C_{\varepsilon }\Delta \varepsilon +C_{T}\Delta T$$

where $C_{\varepsilon }$ and $C_{T}$ represent the sensitivity coefficients for strain and temperature, respectively. The strain sensitivity coefficient was calibrated using a uniaxial tension test, as discussed in [25]. The strain sensitivity coefficient was determined to be 5.43 × 10^−5^ GHz/μɛ. Both ends of the axial-distributed sensor were connected to the Pump and Probe end of NBX-6040 BOTDA. The minimum sampling interval and spatial resolution was 5 cm and 10 cm, respectively. The experimental setup is detailed in Figure 4.

## 4. Results and Discussion

### 4.1. Location of Corrosion Damage of Prestressed Anchor Cables

**Figure 5:**
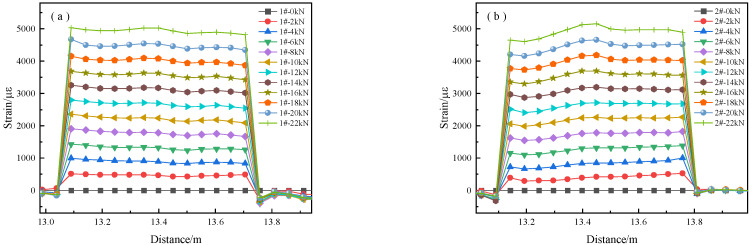

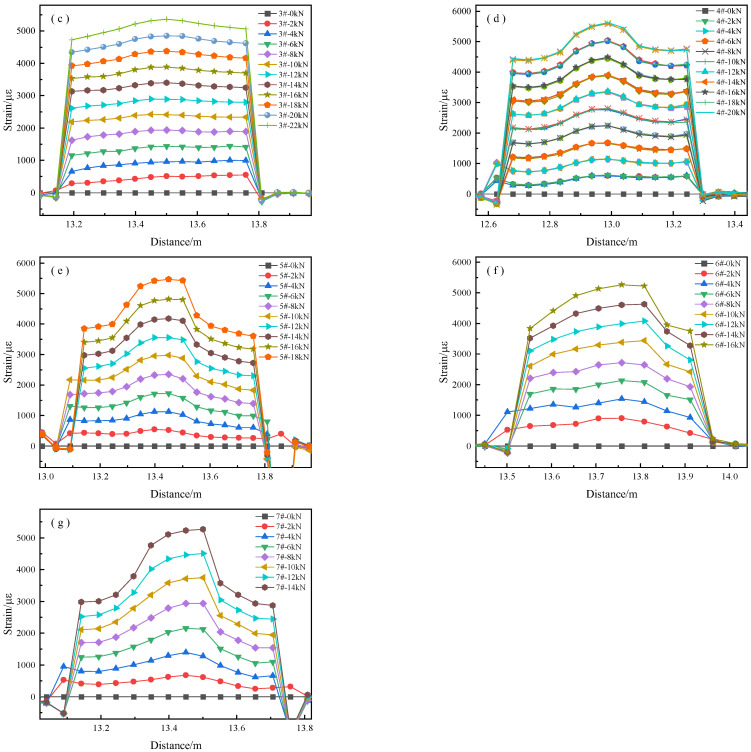
The distributed strain along the specimens with one sectional loss: (**a**) 1#; (**b**) 2#; (**c**) 3#; (**d**) 4#; (**e**) 5#; (**f**) 6#; (**g**) 7#.

Figure 5 shows the distributed strain along the strand. Only the data from the length of the sensor on the strand are plotted. The vertical axis represents the strain determined using the deployed distributed sensor. The horizontal axis represents the length along the installed distributed sensor, starting from the data acquisition system’s pump end. Figure 5a–g represent 1#, 2#, 3#, 4#, 5#, 6#, 7#, respectively, and their section loss rates were 0.48%, 2.45%, 5.20%, 14.24%, 22.92%, 29.26%, 34.87%. We found no peak in Figure 5a–c, while Figure 5d–g had a marked peak. Such a phenomenon is attributable to a small cross-section loss rate; specimens 4#, 5#, 6#, 7# had greater cross-sectional area losses. A greater sectional loss represents a higher corrosion mass loss rate. Hence, the distributed strain depends on the corrosion mass loss rate.

There is a simultaneous strain peak along the strand in Figure 5d–g. The single peak indicates a single corrosion spot on the anchor cable, and its position is basically identical to its actual location along the strand. In addition, the fiber strain is dependent on the section loss rate of these specimens. Hence, the positional accuracy depends on the corrosion sensitivity. The axial distribution fiber monitoring method is thus a feasible way to monitor cable corrosion under high-stress conditions.

### 4.2. Mathematical Model of Corrosion Rate of Prestressed Anchor Cable

**Figure 6:**
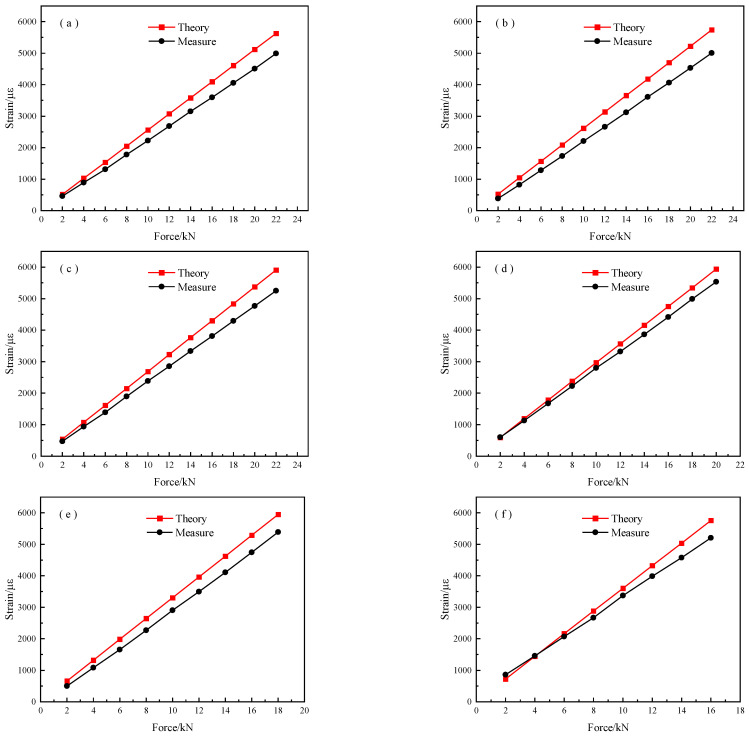

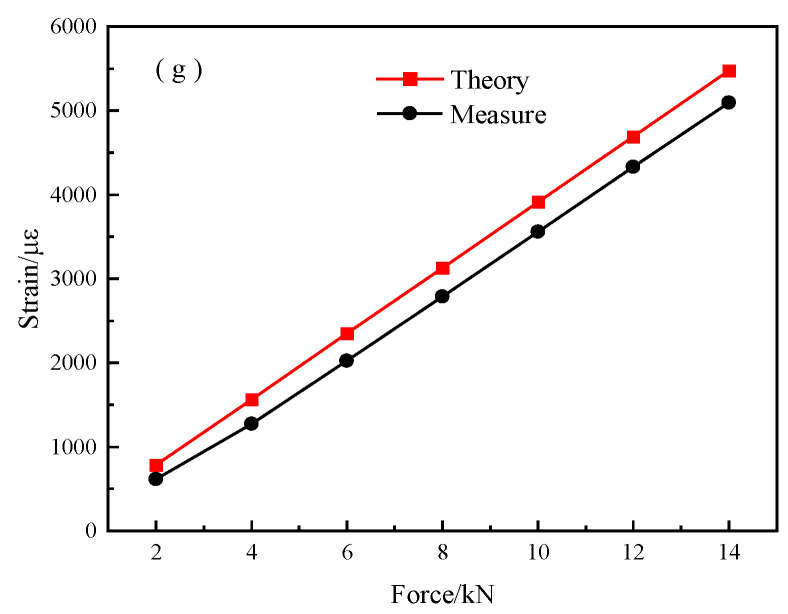
Stress–strain curves of different specimens: (**a**) 8#; (**b**) 9#; (**c**) 10#; (**d**) 11#; (**e**) 12#; (**f**) 13#; (**g**) 14#.

Figure 6 shows the response of the strain to the applied tension. The red curve represents the theoretical strain calculated from Equation (5). The black curve represents the strain measured from an axial-distributed sensor. We compared the strain response to the applied force of the identical specimen under different initial forces, as shown in Figure 6. We found that the theoretical strain is greater the measured strain. Such a phenomenon is obvious under higher stresses. This contributes to the error caused by parameter values, such as elastic modulus E and initial cross-sectional area S0 in Equation (5), which may not be 200 GPa and 19.625 mm^2^ due to manufacturing error. The strand section itself is a random variable due to fabrication error, resulting in a calculable section loss rates error. Moreover, the error depends on the increasing tension. Therefore, the corrosion mass loss rate should not be attained by Equation (5). The parameters of $\varepsilon_{0}$ and ε in Equation (4) are measured by the axial-distributed sensor. The measured values of $\varepsilon_{0}$ and ε are consistent with the actual mechanical parameters of these specimens. It is suggested that the corrosion mass loss rate from Equation (4) is more precise than that of Equation (5). The difference between the theoretical and experimental curves in Figure 6 can be attributed to the inaccuracy of the strain measurements. Such an error is related to the spatial resolution of the distributed optical fiber measurement system used in the experiment. A higher spatial resolution test decreases the predicted corrosion mass rate error.

**Figure 7:**
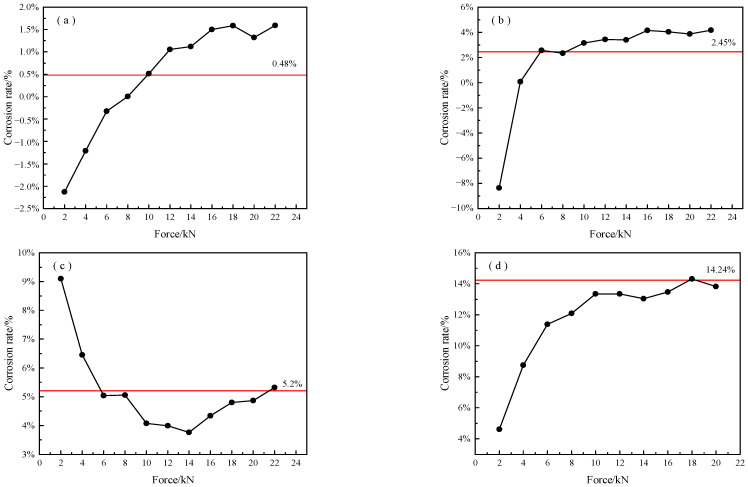

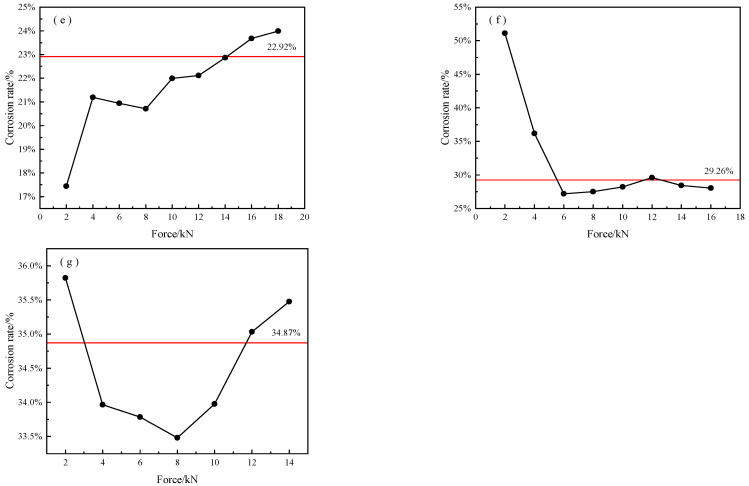
The relation between applied load and fiber strain: (**a**) 1#; (**b**) 2#; (**c**) 3#; (**d**) 4#; (**e**) 5#; (**f**) 6#; (**g**) 7#.

At the same time, based on the above conclusions of corrosion mass loss rate from Equation (4), we used Equation (4) to attain the measured corrosion mass losses of these specimens under different tensions. A ratio in Equation (4) was calculated between two average strain values whereas, in Equation (5), only one strain value was used. Since this value was inaccurate, the corrosion mass loss rate had an error owing to Equation (5). The experimental results are shown in Figure 7. Whether the measured corrosion mass loss rate is correct depends on the tension. A higher prestress results in more precise corrosion mass loss. The red line represents the actual sectional loss rate, which is equal to the corrosion mass loss rate. It was found that the measured corrosion mass loss rate had a lower error when the prestress was more than 12 kN. The measured corrosion rate of specimens no. 1# to 7# were basically consistent with the theoretical corrosion rate fitting curves when the prestress was more than 12 kN. This analysis is in good agreement with the afore-mentioned conclusion of the axial sensor with a greater sensitivity in a higher stress.

**Figure 8:**
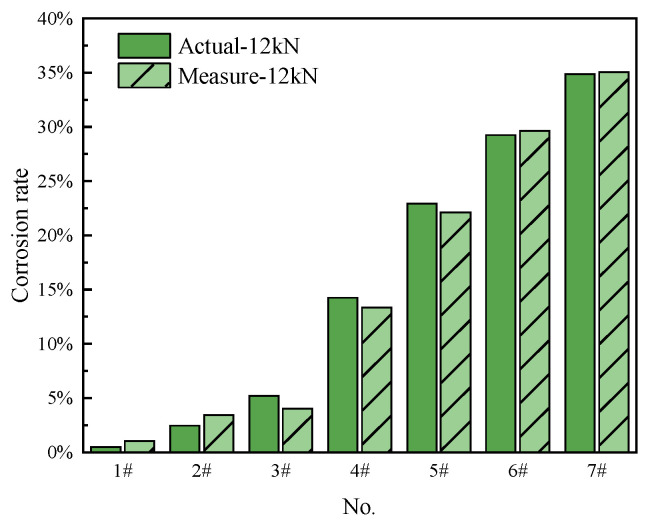
Comparison of actual and tested corrosion mass loss rates.

To summarize, the higher tension and Equation (4) improve the precision of the measured corrosion mass loss rate. The strain data are collected from the non-corroded part of the axial-distributed sensing fiber. The actual calculated corrosion rate is basically consistent with the measured corrosion rate from the collected fiber strain, as shown in Figure 8.

**Figure 9:**
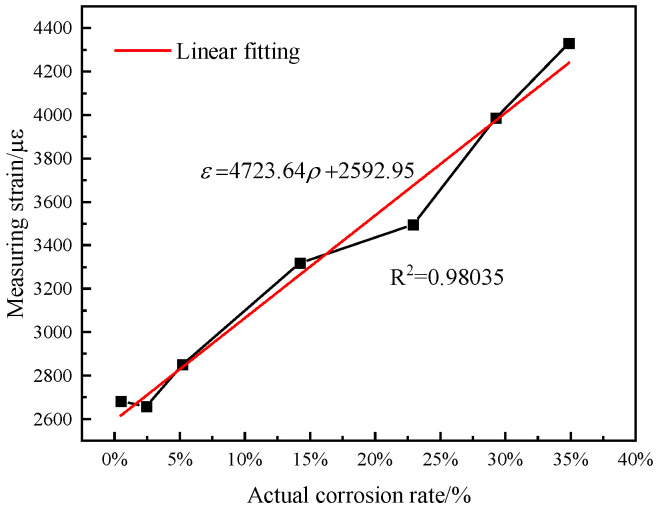
Comparison between tested and actual mass corrosion rates.

Finally, the mathematical model between the corrosion rate and the measured strain from an axial-distributed sensor is acquired. Figure 9 illustrates the distributed strain over the corrosion mass loss rate. The measured strain was generally proportional to the actual corrosion rate. The linear fitting slope of this curve was 4723.46. Its goodness-of-fit score, $R^{2}$, was 0.98035. Hence, the sensitivity of the axial-distributed sensor was 4723.46 με/%, which agrees with the theoretical sensitivity of 4927.18 με/% in Figure 1. Table 2 shows the linear error analysis of the measured corrosion rate, based on Figure 9. We see that specimen 6# has a maximum error of up to 179.43 uɛ.

## 5. Conclusions

This study develops a new method to investigate the corrosion mass loss rate mathematical model with the use of an axial-distributed optical fiber optic sensor. Based on this study, the following key findings can be drawn:

(1) A long-term monitoring method of corrosion damage of prestressed anchor cable is proposed. The results show that the corrosion of the prestressed anchor cable can be monitored by the axial-distributed sensor. Moreover, it has a greater sensitivity when an anchored cable has a higher stress. Its mathematical model relating corrosion mass loss and axial fiber strain is ε=4723.64ρ+2592.95. Therefore, this work provides a feasible method for a real-time and long-term corrosion measurement using the outlined prediction model;
(2) The corrosion length of anchor cable is characterized by the axial distribution fiber strain. The position accuracy depends on its corrosion sensitivity. The axial-distributed optical fiber sensor is used to accurately locate the corrosion damage of the prestressed anchor cable;
(3) The theoretical and experimental curves in Figure 6 can be attributed to the inaccuracy of the strain measurements. The measured strain is related to the spatial resolution of the BOTDA system used for the experiment. The strain measurements presented in the paper underestimated the actual strain. The instrument with a higher spatial resolution is beneficial for attaining the precise strain.

## Figures and Tables

**Table 1:** Structural parameters of different specimens.

No.	Section Depth/mm	Section Loss Rate	Length/cm
1#	0.1	0.48%	20
2#	0.3	2.45%	30
3#	0.5	5.20%	30
4#	1	14.24%	10
5#	1.4	22.92%	20
6#	1.67	29.26%	15
7#	1.9	34.87%	20

**Table 2:** Linear error analysis of measured corrosion rate.

No.	Measured Corrosion Rate Value of Strain	Linear Fitting Value of Strain	Difference Value	Error of Linearity (Difference/Maximum Value)
1#	2682.73	2615.62	67.11	0.0155
2#	2657.38	2708.68	−51.30	−0.0118
3#	2850.94	2838.58	12.36	0.0029
4#	3317.75	3265.60	52.15	0.0120
5#	3496.18	3675.61	−179.43	−0.0414
6#	3985.18	3975.09	10.09	0.0023
7#	4329.10	4240.08	89.02	0.0206

## Data Availability

The data presented in this study are available from the corresponding author, (J.L.), upon reasonable request.
